# Recent Advances in Plant‐Based Dairy Alternatives: Technological Innovations, Nutritional Enhancement, Sustainability, and Consumer Perspectives

**DOI:** 10.1002/fsn3.71590

**Published:** 2026-03-08

**Authors:** Nabeel Ashraf, Zunaira Arshad, Ahmad Din, Huma Bader Ul Ain, Esther Ugo Alum, Tabussam Tufail

**Affiliations:** ^1^ National Institute of Food Science and Technology University of Agriculture Faisalabad Pakistan; ^2^ University Institute of Diet & Nutritional Sciences The University of Lahore Lahore Pakistan; ^3^ School of Food Science and Engineering Yangzhou University Yangzhou China; ^4^ Department of Research and Publications Kampala International University Kampala Uganda; ^5^ College of Pharmaceutical Science Zhejiang University of Technology Hangzhou P.R. China

**Keywords:** fortification, nutritional profile, plant‐based beverages, sustainability, technological advancement

## Abstract

Plant‐based beverages (PBBs) have attracted considerable attention from the global dairy industry as a viable substitute to the milk industry due to their sustainability, nutritional value, and economics. An emerging trend is PBBs, which can be an affordable option for low‐income populations in developing nations and in areas where there is a limited supply of cow's milk. The PBB market is projected to reach a value of $7.3 billion by 2032, growing at a compound annual growth rate (CAGR) of 10.3% from 2023 to $2.8 billion in 2022. The increase of vegans around the world is the main factor propelling the PBB market's expansion. Cereals, legumes, nuts, and seeds are the sources that may be utilized for manufacturing vegetal beverages. The present possibilities and obstacles related to PBB development are explored in this review. This review summarizes novel insights on PBB, the use of novel ingredients, traditional and technologically advanced methods, health benefits, and risks related to consuming these beverages. This comprehensive review also briefly describes the production processes utilized for producing PBBs, the bioavailability of nutrients, their impact on gut microbiota, and the sustainability of PBBs for the circular economy. These insights are intended to assist scientists and food producers in choosing and refining appropriate processing techniques to enhance the nutritional attributes, shelf life, and consumer acceptability of PBBs.

## Graphical abstract

Plant based beverages (PBBs) Raw materials (cereals, nuts, legume, seed and tuber) Market increase: 30 billion USD in 2023 to 160 billion USD by 2030 Protein: cow, quinoa, soy, and a chickpea > 3g Carbohydrate: ↑quinoa, coconut, chickpea, and rice milk Fat: ↑coconut and sesame milk Authentication technologies (For fraud prevention (almonds, pistachios, and cashews) Fingerprinting techniques Raman spectroscopy High‐resolution mass spectrometry Conventional techniques Pasteurization, thermization, and ultra‐high temperature Emerging techniques High hydrostatic pressure, ultrasound, cold plasma and pulsed electric field Health concerns: Microbiological, Mycotoxin, Allergens, Antinutritional Regulatory bodies and labelling European union (EU), Food Safety and inspection Service (FSIS) and Codex Alimentarius commission, Food and drug administration (FDA) Sustainability and environmental impact ↓carbon footprint ↓ land use ↓ global warming potential.

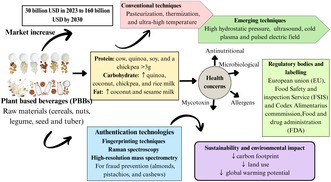

## 1 Introduction

The term dairy substitutes refer to plant‐based dairy alternatives that can be consumed instead of traditional mammalian milk‐based products, especially for people who cannot tolerate dairy products (Moshtaghian et al. 2024). Almost 65% of the population worldwide is lactose intolerant (Bayless et al. 2017). In addition, because they are allergic to milk proteins, many individuals avoid dairy items due to high cholesterol, lifestyle preferences, cost considerations, ethical beliefs, and sustainability concerns (Sethi et al. 2016). Currently, plant‐based beverages (PBBs) are becoming more popular. A number of health‐related factors influence people's decision to choose PBBs. For example, while eating a lot of animal‐based foods raises cholesterol and promotes cardiovascular problems, grains, legumes, seeds, and nuts are good sources of dietary fiber, vitamins, minerals, and antioxidants. According to Aydar et al. (2020), these foods are also classified as functional and nutraceutical foods. Pistollato et al. (2018) demonstrate that plant‐based foods such as nuts and soybeans lower the incidence of cognitive diseases like Alzheimer disorder in addition to its impact on a decline in cardiovascular diseases. The market for plant‐based products is predicted to increase from 30 billion USD in 2023 to 160 billion USD by 2030, showing a significant demand for manufacturing of foods and beverages (Silva et al. 2024). By 2032, plant‐based dairy substitutes are expected to expand at an average yearly growth rate of 9% worldwide (Moshtaghian et al. 2024). A plant‐based food has become more popular recently because of awareness, livestock cruelty, and a need for healthier habits (Sebastiani et al. 2019). A reduction in lactose digestion has been observed in 65% of the global population, as per a study reported in 2020 by the U.S. National Library of Medicine. Lactose intolerance affects 70%–100% of people in East Asia. Plant sources (cereals, legumes, seeds, and nuts) contain antioxidants, micronutrients, and fibers (Tufail et al. 2025a, 2025b). They lowered the chances of heart disease and neurological issues (Pistollato et al. 2018). Because cereals are widely available and reasonably priced, they are the primary component in many plant‐based goods and are recognized as the primary source of micro‐ and macronutrients globally (Gobbetti et al. 2020). Due to their higher antioxidant levels and fatty acid content, PBBs are beneficial since they lower the incidence of diabetes, atherosclerosis, cancer, and cardiovascular illnesses (Zujko and Witkowska 2014). Consumer awareness of food choices and environmental sustainability contributes to increased demand for PBBs (Montemurro et al. 2021).

Plant‐based milk substitutes (PBMS) have seen a rise in sales over the past 10 years and are expected to continue. The global market for PBBs was estimated to be worth $2.8 billion in 2022 and is expected to expand at a compound annual growth rate (CAGR) of 10.3% from 2023 to $7.3 billion by 2032 (Allied Market Research 2023). Many PBBs are widely available in the market and have a long history in every culture (Bernat et al. 2015). Even though some PBBs are deficient in calcium and protein, they are consumed in diets due to their low calorie, lactose, cholesterol, and allergy concerns (Mäkinen et al. 2016). This might raise consumer awareness, raising sales patterns (Jeske et al. 2018). Fermentation is one of the oldest techniques to preserve plant‐derived products. Lowering the amount of sugars and raising quantities of vitamin B_1_, vitamin B_3_, and L‐lysine enhances the finished product's nutrient content and sensory appeal (Rasika et al. 2021). Additionally, 45% more protein in boiling soybeans was absorbed through fermentation, which impacted human health (Ketnawa and Ogawa 2019). Lactic acid fermentation can preserve PBB, which yields organic acid and antimicrobial components (Chinsembu et al. 2015). Fermentation is one way to address the problem of lower bioavailability of vitamins and minerals due to certain anti‐nutrients and polyphenols (Dubey and Patel 2018). Additionally, nutritional profile of PBB is improved via fermentation, which increases the amount of essential protein and vitamins and medicinal properties like anticancer (Costa et al. 2020; Grom et al. 2020).

One practical way to minimize the risk of chronic diseases and provide a complete, nutritious drink for those with or at risk of deficiencies in calcium, vitamin D, and other critical minerals is through the fortification process of PBBs (Pandey et al. 2025). However, there are several negative health impacts associated with PBBs, such as inadequate protein content and poor mineral and vitamin bioavailability. The primary obstacle for nonconventional beverages, particularly those made with legumes, is their complicated processing and unsatisfactory sensory evaluation (Kaur et al. 2026). To address these issues, both conventional and unconventional food processing techniques have been studied to enhance the nutritional content, stability, sensory attributes, and shelf life (Sethi et al. 2016). Due to conventional pasteurization, many heat‐sensitive substances can be lost, and also the quality of the food can be affected due to the off flavor (Barba et al. 2013). To minimize their effects, different nonthermal techniques like HHP (high hydrostatic pressure), HPH (high‐pressure homogenization), ultrasound treatment, and PEF (pulsed electric field) have been contemplated. The scientific assessment of these emerging technologies demonstrated positive outcomes for maintaining nutritional qualities and minimizing the degradation of bioactive substances in PBB (Sethi et al. 2016). This review offers a comprehensive overview of recent advances in PBBs, processing methods, nutritional enhancement, and emerging preservation technologies together with knowledge of sustainability. This review also provides valuable insight into major allergens and contaminants and also the effect of PBBs on gut microbiota and sensory profile.

## 2 Plant‐Based Beverages (PBBs)

**FIGURE 1:**
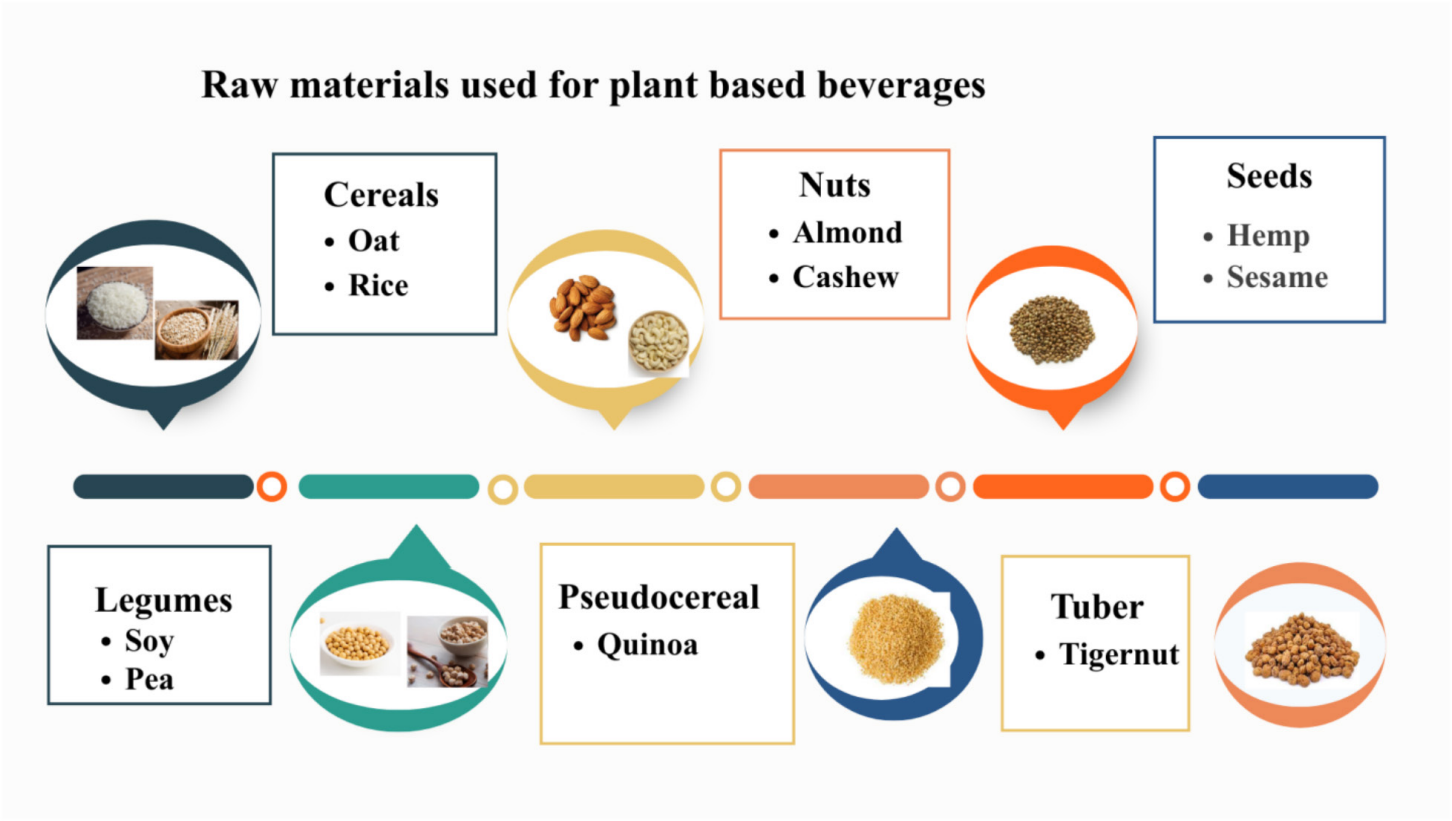
Raw materials of PBBs.

**FIGURE 2:**
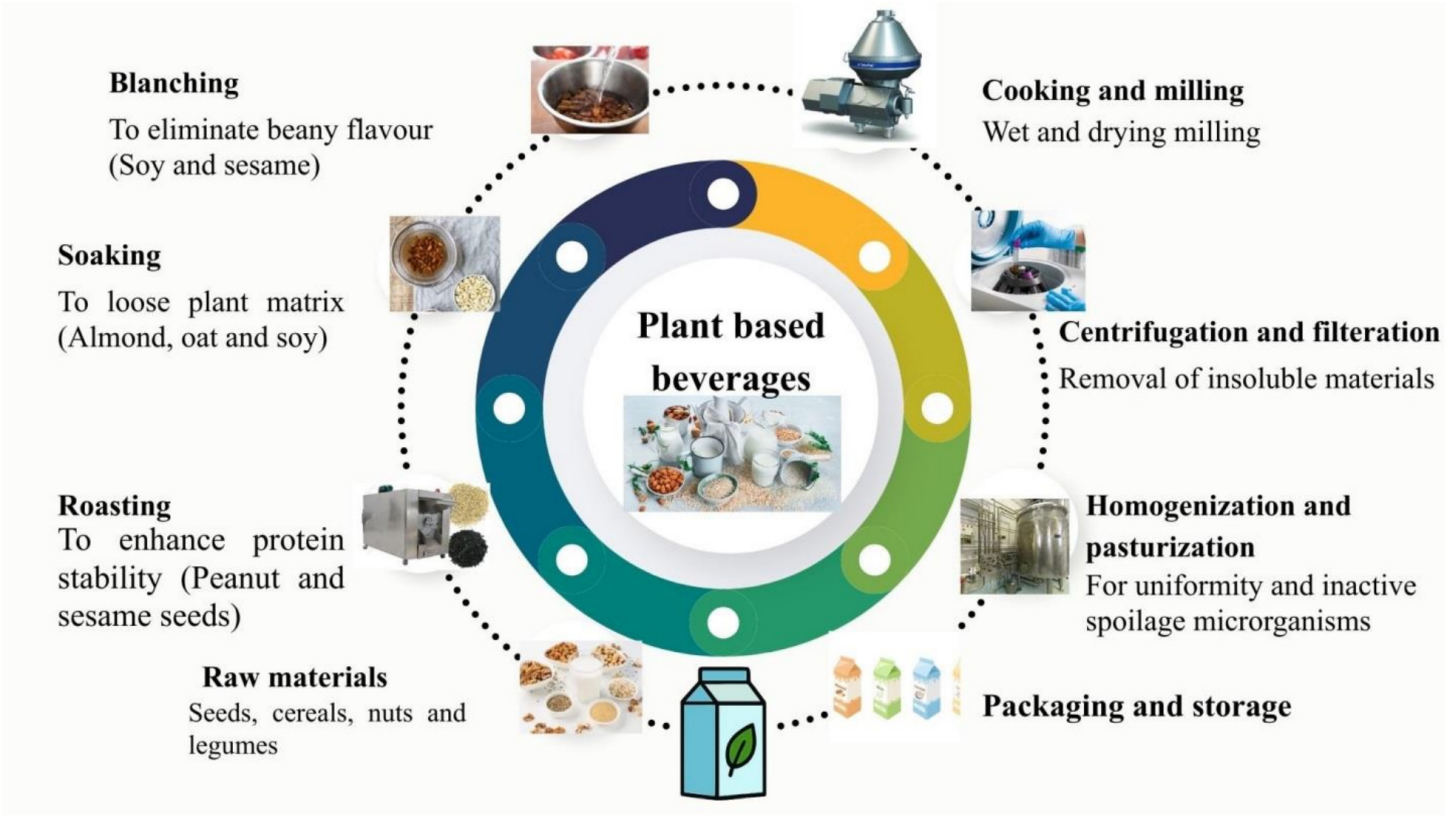
Processing steps of PBBs.

The issue of providing healthy food supplies is becoming a constant concern for companies and consumers, as the number of people who need to be fed rises annually. Raw materials used for the processing of PBBs are shown in Figure 1. Consequently, PBBs have been available for quite some time, and acceptance rises daily due to their increasing sensory appeal (Jeske et al. 2018). Table 1 shows the effect of processing conditions on different types of PBBs. Prior research has explored the possibility that soy products high in protein can lower triglycerides and total cholesterol (Weisse et al. 2010). It has been discovered that fermentation of soy products is significantly effective against diabetes, high blood pressure, cardiovascular disease, and cancerous problems (Jayachandran and Xu 2019). Previous studies showed that in comparison with unfermented soy milk, fermentation of soy milk boosted antioxidant, antibacterial, and anti‐inflammatory properties (Sadeghi et al. 2020; Singh et al. 2020). Early in the 1990s, almond milk was revealed to be free of lactose, cholesterol, and saturated fat. Almond milk is low‐caloric, making it ideal for lactose intolerant individuals (Manzoor 2017a). A recent study found that phenolic contents in almond milk fermented with lactic acid bacteria had antioxidant properties which may lower the risk of diseases linked to oxidative stress (Wansutha et al. 2018). Figure 2 illustrates the processing step for the production of PBBs. Peanut milk is a yellow liquid that is low in fat and high in protein. According to (Arya et al. 2016), it is made by crushing the peanuts with water until the pH reaches 9. The fat is then removed using a cream separator. Lactic acid bacteria (LAB) can ferment peanut milk, yielding a beverage. It is rich in proteins, dietary fibers, minerals, and lipid profiles like phytic and oleic acids. Furthermore, it has been discovered that peanuts contain p‐coumaric acid, known for its positive impacts as an antioxidant (Bansal et al. 2016). Peanuts are abundant in polyphenol compounds like phytosterol, phenolic acid, resveratrol, and flavonoids that have been shown to inhibit the digestion of lipids from consumed food (Arya et al. 2016). Oat b‐glucan has been shown to have anticancer properties, as it significantly lowers blood pressure and blood cholesterol levels while reducing substances that cause colon cancer (Rasane et al. 2015). However, the value of oats in a daily diet is further increased by essential amino acids, such as linoleic acid (36.2%–40.4%) (Sterna et al. 2016). Additionally, it contains vitamins, phenolic compounds, and other micronutrients with various biological and therapeutic properties, like antitumor and antioxidant properties (Babolani Mogadam et al. 2022).

**TABLE 1:** Effect of processing conditions on different types of PBBs.

Raw materials	Processing conditions	Results	References
Soy	Water soak for 16 h at 20°C, blanched with 1.25% NaHCO3 for 30 min, wet milled for 10 min and filter it	Blanching deactivates lipoxygenase enzyme, enhances nutritional status, rich in protein and fiber	Basharat et al. (2020), De et al. (2022)
Oat	Soak in water for 1, 6, and 8 h. The enzyme was introduced into the slurry for 49 min at 75°C and filters it. Enzyme deactivated by heating 5 min at 100°C	Heat inactivates enzymes, and starch is hydrolyzed by amylase. Oat milk is lactose‐free and nutrient rich and phytochemicals	Syed et al. (2020), Yu et al. (2023)
Peanut	Roasted at 225°C (25 min), soaked at 4°C (6 h), nuts drained, rinsed, and wet milled (5 min), homogenized as well as pasteurized (121°C for 15 min)	Protein isolates and emulsion stability increased by roasting. Higher protein and minerals in comparison to cow milk	Zaaboul et al. (2019)
Sesame	Seeds roasted (145°C for 20 min), washed with water, blanched at 95°C, homogenized at 55°C for 6 MPa then pasteurized for 30 min and filter it	Enhancing the overall taste and texture of the milk while reducing the bitterness and undesirable flavors	Ahmadian‐Kouchaksaraei et al. (2014), Kongkachad and Puttongsiri (2017)
Almond	Soaked at 4°C, blanching at 85°C (30 min), wet milling at 18,000 rpm (2 min), homogenized at 350 MPa (UHP), 85°C	Toxins are released into water. Almond milk has fewer calories and higher nutrients	Alozie et al. (2015), Maria and Victoria (2018)
Cashew	De‐hulled and soaked in hot water (4°C: 5 h), make a slurry and pasteurized (120°C–122°C: 15 min) then store in plastic bottles (4°C)	High in calcium, zinc, and iron and higher nutrient than soy milk	Manzoor (2017b)
Coconut	After cracking the coconuts and heating them to 55°C, the thick slurry was filtered to extract the coconut milk, which was then pasteurized at 62.8°C (30–60 min)	Because coconut milk has very little lactose and cholesterol, it can be consumed by vegans and lactose intolerance people	Tulashie et al. (2022)
Hemp	Dehulled hemp seeds were soaked in water (1:15), heated to 47.34°C, blended for two to 4 min, and then filtered. Pasteurization at 73°C, homogenization at 7500 rpm, storage, and cooling	The results showed that heating the hemp seed milk extract for 60 min at 48°C with the use of a water bath increased its fat and protein content	Thakur et al. (2024)
Tiger nut	Dried, sorted, cleaned, and then allowed to soak for 24 h at 30°C in tap water at a 1:3 (w/v) ratio. Mix with distilled water (1:3 w/v) after blending for 5 min. After filtering the homogenous slurry, it was squeezed until no extract remained. After being heated to 70°C (20 min), then cooled to 4°C	Tiger nut milk offers vegetarians and other consumers a safe PBB to cow's skim milk since it is high in phenolic contents which linked to potent antioxidant action	Shalabi (2023)
Chickpea	After soaking for 12 h, it was cooked for 20 min at 120°C and 2.0 atm in 2.5 L of water before being filtered through a cloth. The ratio of 1:3 (1 coconut to 3 boiling water) was used to prepare the coconut extract, which was then blended for 2 min at medium speed and filtered through a cloth to produce the liquid form	The results demonstrated a superior nutritional profile (such as protein and fat content) when compared to cow's milk and other plant‐based beverages. Nonetheless, the new PBB has greater amounts of calcium and protein	Rincon et al. (2020)
Quinoa	Cleaning, sorting, dehulling, and soaking the quinoa in a 1:3 solution of 0.5%–1.0% sodium bicarbonate at room temperature. After soaking, the original quinoa's weight doubles. The split, or dehulled beans, are then mashed in hot water at a ratio of 1:7 and filtered to produce milk	Quinoa milk is high in protein, low in fat and carbs, and cholesterol‐free. Babies, children, the elderly, and expectant or nursing mothers all benefit greatly from this beverage	Zafar et al. (2018)

Li and Lu (2015) studied that sesame can be referred to as the “crown of eight grains” and is a nutrient‐rich grain that is a “multipurpose nutritional store.” Due to its higher calcium level, relatively low cholesterol level, and vital amino acid content, it can also serve as an alternative to mammalian milk (Dharini et al. 2023). Small levels of tannins and the anti‐nutritional elements like phytic acid and oxalic acids are the primary constituents of sesame seeds. Additionally, coconut milk is high in nutrients and minerals like potassium, sodium, calcium, magnesium, and iron. Apart from its antiviral, antibacterial, and antitumor effects, consuming coconut milk is associated with health benefits. Furthermore, it contains lauric acid and saturated fat, which are present in mother's milk and have been linked to mental development (Tulashie et al. 2022).

Hemp seeds are abundant in vital nutrients like zinc, magnesium, iron, sulfur, potassium, calcium, iron, and phosphorus, alongside carbohydrates (20%–30%), oil (24.5%–35.5%), carotenoid and sulfur‐rich proteins (20%–25%), and proteins like arginine. The hemp seeds possess various biological properties, including potential blood pressure‐lowering effects and antioxidant activity (Ustun‐Argon 2019; Wang et al. 2018). Tiger nuts have long been known for their nutritional and health‐related advantages. It has about 10% protein, 50% digestible carbs, and 9% dietary fiber, including cellulose and lignin (Munekata et al. 2021). A unique nutritious beverage, chickpea milk has no cholesterol and is high in proteins, carbs, and isoflavones. Unlike other PBBs, this one does not cause allergic responses (Zhang et al. 2022). Because it can lessen the environmental impact of cattle production, chickpea milk has drawn significant attention. Chickpea milk is a darker color and has a more noticeable yellow hue than mammalian milk. One of its unique qualities is its beany flavor, which is typical of numerous products made from legumes that can be eliminated through fermentation, boiling, germination, and other processing methods (Rincon et al. 2020). Quinoa seeds have more protein than most cereals but less than legumes. Quinoa seeds are a type of gluten‐free grain. More energy, fiber, calcium, phosphorus, and iron can be found in quinoa seeds than in regular cereals. The powerful advantages derived from polyphenols are linked to the activity of antioxidants, which function as anticarcinogenic and immunological resistance against cancer (Salwa 2019).

## 3 Nutritional Values of PBBs

A growing number of people are experiencing dietary constraints, driving up consumer preference for dairy free milk substitutes. The kind and amount of the ingredients, the manufacturing process, and the additional components all have an impact on the nutritional content of PBBs (Rachtan‐Janicka et al. 2025). Table 2 shows the nutritional value of PBBs in comparison with cow milk. According to Clay et al. (2022), some PBBs have arisen, such as soybean, almond, oat, peanut, and sesame. High‐quality proteins can be produced at a minimal cost by using soy milk. Furthermore, in comparison with cow milk, it contains a substantial quantity of calories (51.7/100 g), proteins (3.89 g/100 g), and lipids (2.39 g/100 g) (Mazumder and Begum 2016).

**TABLE 2:** Nutritional values of PBBs.

Nutritional profile	Milk types (Plant and cow)	References	References	References	References
Protein	Cow	4.37 (g/100 g) (Silva et al. 2024)	8.2 (g) (Ramsing and Santo 2023)	2.81 (%) (Matejic et al. 2023)	
	Cashew	1.8 (g) (Ramsing and Santo 2023)	0.42 (g/100 mL)		
	Almond	1.54 (g) (Sunidhi et al. 2021)	0.58 (g/100 mL)	0.08 (%) (Matejic et al. 2023)	1.67 (g) (Vanga and Raghavan 2018)
	Rice	0.7 (g) (Ramsing and Santo 2023)	0.28 (g/100 mL)	1.85 (g) (Thuy et al. 2020)	0.36 (g/100 mL) (Pérez‐Rodríguez et al. 2023)
	Sesame	1.5 (g) (Sunidhi et al. 2021)	2.65 (%) (Vahini and Mary 2021)	2.52 (%) (Quasem et al. 2009)	
	Soy	6.1 (g) (Ramsing and Santo 2023)	7 (g) (Sunidhi et al. 2021)	1.8 (%) (Matejic et al. 2023)	3.1 (g/100 mL) (Pérez‐Rodríguez et al. 2023)
	Oat	2.7 (g) (Ramsing and Santo 2023)	2.5 (g) (Sunidhi et al. 2021)	3–3.5 (g/100 mL) (Le et al. 2025)	1.76 (%) (Pallavi et al. 2022)
	Peanut	3.91 (%) (Abou‐Dobara et al. 2016)	3.8 (%) (Jain et al. 2013)		
	Coconut	2.90 (g/100 g) (Tulashie et al. 2022)			
	Tiger nut	1.58% (Shalabi 2023)			
	Hemp	0.83%–4%			
	Quinoa	4.5 g (Zafar et al. 2018)			
	Chickpea	3.54 (g/100 mL) (Seifu et al. 2025)			
Carbohydrate	Cow	4.78 (g/100 mL)	11.71 (g) (Sunidhi et al. 2021)	4.9 (g/100 mL) (Antunes et al. 2025)	
	Cashew	3.75 g/100 mL	3.74 (g/100g) (Silva et al. 2024)		
	Almond	0.58 (g/100 mL)	1.52 (g) (Sunidhi et al. 2021)	1.32 (g) (Pérez‐Rodríguez et al. 2023; Vanga and Raghavan 2018)	1.9 (g/100 g) (Sterup Moore et al. 2024)
	Rice	9.17 (g/100 mL)	57.3 (g/100 g) (Sunidhi et al. 2021)	11.2 (g/100 mL) (Pérez‐Rodríguez et al. 2023)	12.7 (g/100 mL) (Sterup Moore et al. 2024)
	Sesame	4.69 (g/100 g) (Afaneh et al. 2011)			
	Soy	1.67 (g/100 mL)	4.23 (g) (Sunidhi et al. 2021)	1.58 (g/100 g) (Silva et al. 2024)	2.6 (g/100 mL) (Pérez‐Rodríguez et al. 2023)
	Oat	16 (g) (Sunidhi et al. 2021)	2 (g) (Sunidhi et al. 2021)	8.3 (g/100 mL) (Pérez‐Rodríguez et al. 2023)	7.95 (g/100 mL) (Sterup Moore et al. 2024)
	Peanut	4.7 (%) (Yadav et al. 2018)			
	Coconut	5.5 g/100 g (Tulashie et al. 2022)			
	Tiger nut	3.61% (Shalabi 2023)			
	Hemp	0.3%–20%			
	Quinoa	5.0 g (Zafar et al. 2018)			
	Chickpea	8.57 (g/100 mL) (Seifu et al. 2025)			
Fat	Cow	3.27 (g/100 mL)	7.93 (g) (Sunidhi et al. 2021)		
	Cashew	1.04 (g/100 mL)	3.6 (g/100 g) (Silva et al. 2024)		
	Almond	1.10 (g/100 mL)	2.88 (g) (Sunidhi et al. 2021)	2.7 (g) (Vanga and Raghavan 2018)	2 (g/100 mL) (Pérez‐Rodríguez et al. 2023)
	Rice	0.97 (g/100 mL)	1 (g/100 mL) (Pérez‐Rodríguez et al. 2023)	0.45 (g/100 g) (Sterup Moore et al. 2024)	
	Sesame	7.82 (g/100 g) (Ahmadian‐Kouchaksaraei et al. 2014)			
	Soy	1.67 (g/100 mL)	3.91 (g) (Sunidhi et al. 2021)	2.6 (g/100 g) (Silva et al. 2024)	1.8 (g/100 mL) (Pérez‐Rodríguez et al. 2023)
	Oat	5 (g) (Sunidhi et al. 2021)	2 (%) (Pallavi et al. 2022)	0.76 (g/100 g) (Sterup Moore et al. 2024)	
	Peanut	4.5 (%) (Abou‐Dobara et al. 2016)	5 (%) (Elsamani 2016)		
	Coconut	23.8 g/100 g (Tulashie et al. 2022)			
	Tiger nut	1.72% (Shalabi 2023)			
	Hemp	1.25%–4.61%			
	Quinoa	2.8 g (Zafar et al. 2018)			
	Chickpea	0.94 (g/100 mL) (Seifu et al. 2025)			
Ash	Cow	0.67 (g/100 mL)	0.72 (g/100 g) (Sterup Moore et al. 2024)		
	Cashew	0.2 (g/100 g) (Silva et al. 2024)			
	Almond	0.68 (g/100 mL)	0.15 (g/100 g) (Sterup Moore et al. 2024)		
	Rice	0.30 (g/100 mL)	0.09 (g/100 g) (Sterup Moore et al. 2024)		
	Sesame	0.48 (%) (Afaneh et al. 2011)			
	Soy	0.3 (g/100 g) (Silva et al. 2024)	0.46 (g/100 g) (Sterup Moore et al. 2024)		
	Oat	0.8 (%) (Pallavi et al. 2022)	0.18 (g/100 g) (Sterup Moore et al. 2024)		
	Peanut	0.16 (%) (Jain et al. 2013)			
	Coconut	0.7 (g/100 g) (Tulashie et al. 2022)			
	Tiger nut	0.61% (Shalabi 2023)			
	Hemp	0.47%			
	Quinoa	—			
	Chickpea	85.23 (g/100 mL) (Seifu et al. 2025)			

Almonds greatly reduce the incidence of cardiac issues because they are high in fatty acids, minerals, and polyphenols (Manzoor 2017a). Almond milk also contains other nutrients, including zinc, potassium, phosphorus, magnesium, and calcium (Vanga and Raghavan 2018). Peanuts and peanut milk products are exceptionally rich in amino acids, minerals, and essential lipids like oleic and linoleic acids, which are highly valued in human nutrition (Bensmira and Jiang 2012). According to Gamli and Atasoy (2018), peanut milk has more fat, protein, and calories than cow's milk. The phenolic substances, avenanthramides, saponins, phytic acid, sterols, and numerous other nutrients found in oat milk contribute to antioxidant properties. Oats include significant levels of starch, about 60%, and somewhat balanced amounts of protein ranging from 11%–15% and fats (5%–8%). In addition, oats contain 0.54% calcium and range from 2.3 to 8.5 dietary fibers (Rasane et al. 2015). The benefits of calcium, lipids, and protein can be obtained by incorporating sesame into milk or pastes. On the other hand, when it comes to protein content, sesame seeds are the most underestimated. Methionine and cysteine are abundant in sesame protein, which is also extremely heat stable (Dharini et al. 2023). Soymilk is more protein‐rich than milk from mammals, but it lacks the lactose or sugar needed for bacterial growth; soymilk supplemented with lactose or glucose meets the requirements for lactic acid fermentation (Kim and Park 2012). It was discovered that using dairy strains to ferment cashew milk improved the end product's flavor, creating new possibilities for using LAB for plant‐based milk fermentation (Shori and Al Zahrani 2021). The protein content of rice milk is insufficient (Mori et al. 2015). A milk substitute that can be used to make kefir is rice milk. LAB also aids in the fermentation of rice milk, resulting in the production of cheese and yoghurt (Fawzi et al. 2022).

## 4 Fortifications of PBBs

Oat, almond, coconut, rice, and soy milk are among the most significant segments of this growing industry for plant‐based substitutes for cow's milk (Aydar et al. 2020). Concerns have been raised over their nutritional characteristics despite their well‐established ethical and environmental advantages (McClements 2020b). For instance, a lot of PBBs lack essential minerals (such as calcium and vitamin D), which might cause long‐term health issues if not acquired in adequate amounts from different sources (Aydar et al. 2020). Therefore, one effective method of preventing deficiency in these micronutrients may be the nutritional fortification of PBBs (Zhou et al. 2021). The fortification of various PBBs varieties is shown in Table 3. Size‐reduction techniques that use mechanical, chemical, and/or enzymatic means to break down the original plant tissue structure are commonly used to produce PBB (McClements 2020a). This results in the release of the oil bodies in cashews, coconuts, oats, soybeans, and almonds. These oil components are colloidal substances with a phospholipid/protein coating covering a triglyceride‐rich core (Nikiforidis 2019). The botanical origin of the seeds determines the fatty acid (FA) structure of the oil components. For example, the oil bodies of soybeans are mostly made up of polyunsaturated long‐chain FA, while those of coconuts are primarily made up of medium‐chain saturated FA. The oil bodies' surface phospholipid/protein monolayer affects their colloidal stability, including the ability to assemble under various environmental circumstances (Wang et al. 2019). Since the majority of the triacylglycerol‐rich core of oil bodies is hydrophobic, hydrophobic nutraceuticals can be accommodated there. These health‐promoting bioactive substances can therefore be added to PBBs (McClements 2020b). However, due to the presence of a hydrophilic aqueous phase around them, it is frequently difficult to incorporate highly hydrophobic molecules into an oil body solution. In a prior investigation, we demonstrated that the pH‐driven loading technique could be used to incorporate curcumin into the oil bodies in soymilk, improving its in vitro bioaccessibility and storage stability (Zheng et al. 2021). Furthermore, both the soymilk (59%) and the cow milk (40%) made using the pH‐driven approach had significantly higher curcumin bioaccessibilities (Zheng et al. 2021).

**TABLE 3:** Fortifications of PBBs.

Beverages types	Fortifications	Results	References
Cashew + almond + oat + coconut milk	Curcumin encapsulated using simple pH‐driven method	Higher encapsulation and bio accessibilities in all PBBs and higher sensory acceptance rate	Zheng et al. (2021)
Almond milk	Calcium fortification (CaCl_2_ and CaCO_3_)	↑ calcium bioaccessibility in calcium carbonate and useful for nutritional fortified milk	Zhou et al. (2021)
Rice milk	Hydrolyzed peanut and calcium hydroxyl phosphate nanoparticles	↑ antimicrobial properties and ↑ nutritional profile	Abdel Hamid et al. (2020)
Oat milk	Pea and potato protein isolate	1% of pea and potato protein has significant effect on sensory and volatile profile	McCarron et al. (2024)
Soy milk	Vitamin E, soy protein isolate, and calcium	↑ nutrient, ↑ bioactive compounds and higher consumer acceptability	Taha et al. (2024)
Almond and soy milk	Chia and sesame seed	↑ mineral, ↑ carbohydrate contents	

Popular PBBs are soy milk and almond milk. Adding chia and sesame seeds to soymilk and almond milk to increase their nutritious content is the aim of this endeavor. Chia and sesame seeds were chosen due to their high‐quality proteins and affordability. According to (Ramsing and Santo 2023), this fortification strategy might be advantageous, especially for people who are vegan or lactose intolerant. While soymilk and almond milk are good sources of protein for vegans and lactose intolerant people, they do not provide all of the necessary amino acids (Katunzi‐Kilewela et al. 2021).

## 5 Effects of PBBs on the Gut Microbiota (GM)

The gut microbiota is characterized by certain microbial species that are more common in the gut environment and their specific presence and function (Gallo et al. 2024). The phyla Firmicutes and Bacteroidetes make up as much as 90% of the overall makeup of the healthy gut microbiota (Tian et al. 2022). Vegetable beverages are recognized as functional foods despite having fewer nutrients than dairy milks due to their phytonutrient‐driven nutritional advantages (Kehinde et al. 2020). Consequently, PBBs might be the best way to give probiotics to people who are lactose intolerant or allergic to milk proteins (Cakebread et al. 2022). Fermented vegetable beverages can serve as a substitute for fermented milk in order to satisfy consumer demand (Ziarno et al. 2019). The majority of traditional PBBs are produced by spontaneous fermentation, which shortens their shelf life (Rasika et al. 2021). Fermented items may benefit from the addition of probiotics and starter cultures (Freire et al. 2017). Almond extract is a major source of vitamins, particularly vitamin E (alpha‐tocopherol). This potent antioxidant must be obtained through diet or supplements since the body is unable to produce it. Almonds can reduce serum cholesterol levels and may have prebiotic qualities (Sethi et al. 2016). Furthermore, a variety of Lactobacillus and Bifidobacterium strains can be used to ferment almond xylooligosaccharide (XOS). It comprises fiber and polyphenols that encourage microbial fermentation, changing the gut microbiota's makeup (Zahrani and Shori 2023).

Coconut milk may be the perfect fermentative source to promote probiotic growth, according to the development of all these products and the assessment of probiotic survival under refrigeration temperatures for 21 to 28 days. The survival of probiotics is not adversely affected by the addition of components like tapioca (Vitheejongjaroen et al. 2024). A coconut beverage that was kept for 30 days showed that the viability of *Lb. reuteri* DSM 17,938 and *Lb. reuteri* LR 92 was reduced by 0.8% and 6.1%, respectively (Mauro and Garcia 2019). Oats are a dependable supply of β‐glucan, which benefits intestinal health in humans (Benmeziane and Belleili 2023). Chen et al. (2020) found that the viability of a fermented beverage enhanced with *Lb. fermentum* remained steady up to 14 days during storage. The capacity of probiotics to proliferate in a homemade oat extract in comparison to its commercial version was examined by Łopusiewicz (2023). When considering the number of bioactive components and antioxidant activity, they found that produced oat milk demonstrated a more favorable profile. When making probiotic products, soy extract is frequently used as a vegetable source (Rana et al. 2024). According to Costa et al. (2017), a fermented beverage had fewer than 10^6^ CFU/g of *Lb. acidophilus* and Bifidobacterium subspecies because it lacks enough vitamins and amino acids.

Soy beverages have received a lot of attention when it comes to their impact on GM (Fujisawa et al. 2017; Sadeghi et al. 2020), whereas others, such as almond beverages, have not gotten as much attention (Cakebread et al. 2022). Because oats include a variety of dietary fiber and polyphenol content, ingesting them can help the colon produce Short‐chain fatty acids (Fabiano et al. 2025). On a global scale, the GM's outcomes following the administration of vegetable beverages might be deemed advantageous (Fujisawa et al. 2017; Inoguchi et al. 2012). For lactobacilli, the same outcome was found (Cakebread et al. 2022; Gurung et al. 2023; Lee et al. 2015). According to a prior study, soy protein administration led to positive modifications in the GM when compared to cow milk (CM) protein ingestion (Sadeghi et al. 2020). However, some outcomes cannot be advantageous to the host. For instance, piglets fed a plant‐based formula showed an increase in *Fusobacterium* and *Salmonella enterica* (Gurung et al. 2023). A genus of bacteria called *Fusobacterium* is linked to the occurrence of colon cancer (Qin et al. 2024). Additionally, a rise in the ratio of Firmicutes to Bacteroidetes was noted (Lee et al. 2015), which could be advantageous for GM. Since a decrease in GM was shown following soymilk consumption in other studies, the increase in Proteobacteria appears inconsistent (Fujisawa et al. 2017). Finally, after consuming soymilk in a mouse model, no appreciable modifications in the GM were seen in other studies (Wang et al. 2013). When the results for vegetable beverages were compared, it was discovered that almond milk ingestion produced a greater relative abundance than soymilk in a rat model (Cakebread et al. 2022). It is interesting to note that the same study's findings on bone density prevention revealed comparable outcomes for CM, soy, and almond beverages (Cakebread et al. 2022). With a few notable exceptions—increases in *Fusobacterium* or *Salmonella enterica* —the majority of the results were positive. According to the authors, these increases were slight and only made up a small amount of the bacterial groups in the GM (Gurung et al. 2023).

## 6 Effect of Emerging Techniques on PBBs

The production of PBBs presents significant technological challenges, particularly in ensuring physical stability while minimizing reliance on additives. Emerging processing technologies, including ultrasound, PEFs, ohmic heating, and various homogenization techniques, offer promising solutions by inactivating microorganisms, reducing particle size, and improving viscosity (Alehosseini et al. 2025). Various microorganisms may naturally arise during processing, thus heat treatments must be carried out. To eliminate the harmful germs and render PBBs safe for consumption, a number of thermal techniques have been used, including pasteurization, thermization, and ultra‐high temperature (UHT). Pasteurization is the thermal procedure that keeps at (72°C: 15 min) and then quickly cools it down. Thermization reduces the microbial spoilage load of milk by heating it to 63°C for 5 s. UHT involves heating the product for 5–10 s at a temperature between 135°C and 150°C (Zandona 2021). Plant based milk products use different heat treatment parameters. It should be mentioned that differing materials and additives may cause variations in processing time and heat temperature. Heat treatment is typically used to reduce antinutrients such as trypsin inhibitors in addition to inactivating bacteria. Heat treatment may lessen the soymilk's trypsin inhibitory action (Romulo 2022a, 2022b). Trypsin inhibitors are undesirable because they may interfere with the absorption of proteins and lower their bioavailability (Aydar et al. 2020). It has been found that additional heating methods, including steaming and blanching, inactivate enzymes (lipase + lipoxygenase), and raise the yield of total solids and protein. According to Popova and Mihaylova (2019), overheating PBBs resulted in the breakdown of amino acids, a reduction in nutritional content, and other detrimental responses. Heat treatment is in charge of starch gelatinization and the formation of the product's gel consistency in PBBs with high starch content, like cereal‐based milk, which lowers consumer acceptability (Silva et al. 2020).

**FIGURE 3:**
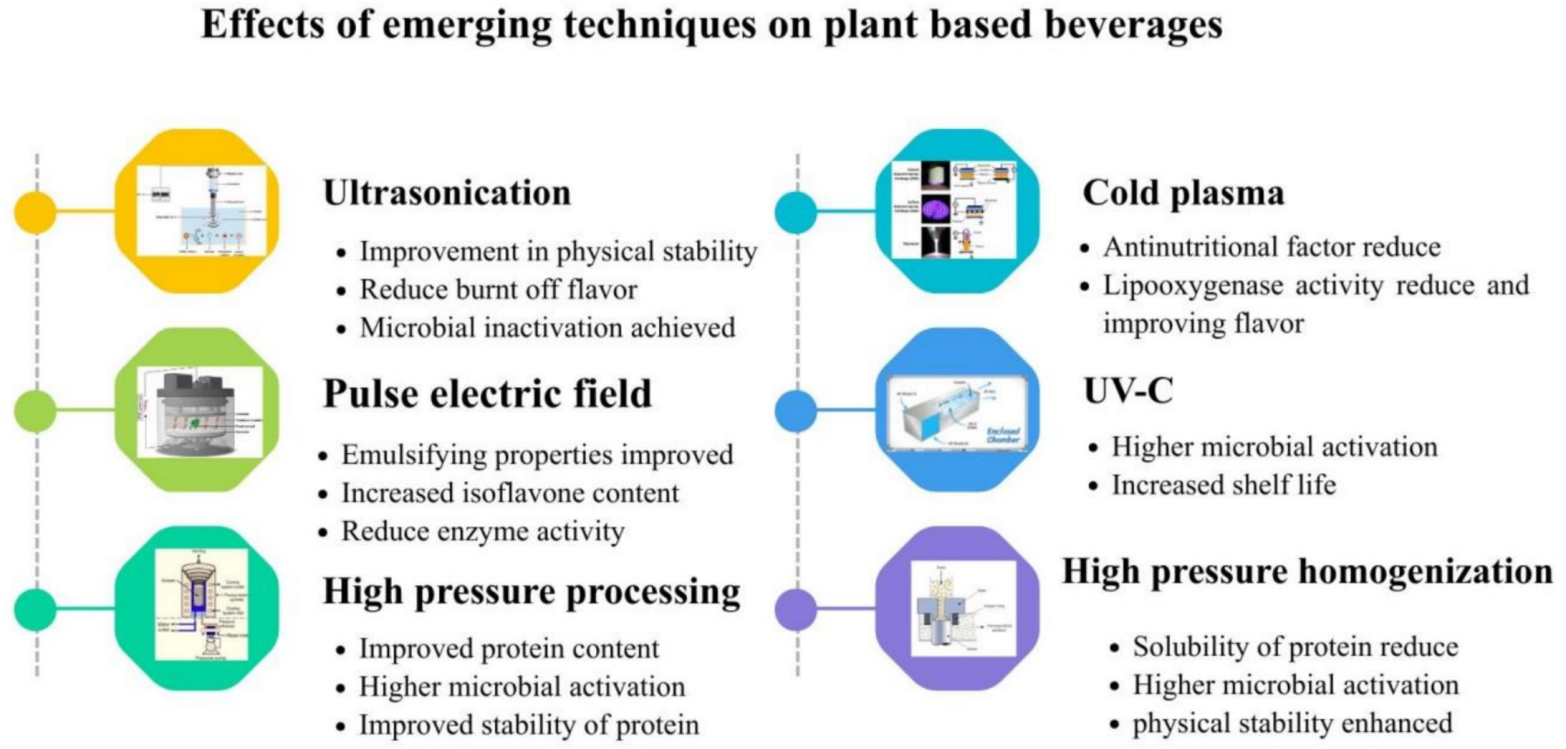
Effect of emerging techniques on different types of PBBS.

Traditional pasteurization processes have the potential to cause adverse reactions and degrade heat‐sensitive components, thereby affecting physicochemical properties and contributing to unpleasant flavor (Barba et al. 2013). To overcome these limitations, new technologies including pulsed electric fields, high hydrostatic pressure, high‐pressure homogenization, and ultrasound are used. The scientific assessment of these emerging technologies demonstrated favorable outcomes in enhancing shelf life and preserving nutritional properties in plant‐based products (Sethi et al. 2016). Figure 3 shows the effects of emerging techniques on PBBs.

To enhance the stability and physicochemical properties of PBBs emulsions while minimizing the impact on their nutritional values, an alternative food processing technique called high‐pressure homogenization (HPH) can be employed (Bernat et al. 2015). The impact of emerging technologies on quality parameters of different types of PBBs (Table 4). By the reduction of colloidal particle size and the generation of more evenly sized particles, the high‐pressure technique may optimize the consistency associated with PBBs (Valencia‐Flores et al. 2013). Soymilk's beany flavor can be subdued with ultra‐high‐pressure homogenization (UHPH) (Poliseli‐Scopel et al. 2013). Compared to soymilks processed using a different method (pasteurization and UHPH at 200 MPa: 55°C), the soymilks treated with UHPH (200 MPa: 75°C) had lower grass and beany sensory ratings. Research has demonstrated several interesting ways to remove the off flavors from PBBs (Tripathi et al. 2015), but further work is needed to refine the actual structure and understand how different methods and technologies work. One crucial aspect of PBBs is their product stability. Colloidal particles tend to settle to the bottom of storage containers, rendering PBBs unstable. Particle dispersion and precipitation primarily affect the sensory qualities of PBBs, which may show diminished ratings in terms of odor, consistency, and flavor (Gul et al. 2018). The sedimentation formation of particles during storage was prevented using UHPH technology, which operates at 200 MPa at 55°C and 75°C (Poliseli‐Scopel et al. 2013). Although UHPH resulted in a more stable product, pasteurization enhanced dispersed particles' stabilization immediately following processing and throughout storage. Soymilk treated with calcium and heated with hydrophilic polymer had a comparable result during processing (Manassero et al. 2016). Samples subjected to thermal treatment (80°C and 90°C at 0.1 MPa) and prepared with 15 mmol Ca/L exhibited a rise in particle sedimentation after 5 days of storage as a result of calcium addition.

**TABLE 4:** Effect of emerging processing on the preservation of PBBs.

Techniques	Product (conditions)	Effects	References
Ultrasonication	Soy milk: Microwave roasting and US frequency (20 kHz, 130 W: 3–9 min)	Reduce beany flavor and better retention of bioactive compounds	Lee and Lee (2023)
Ultrasonication	Soymilk; Frequency (24 kHz) amplitude 50%, 75%, 100% at temperature (30°C, 45°C, 60°C) time (20, 40, 60 min)	Increase in isoflavone concentration	de la Morales‐ Peña et al. (2018)
Pulsed electric field	Oat pea beverage; Pre‐heating (30°C–45°C), electric field strength (8.2–10.4 kV/cm) and specific energy input (77–244 kJ/L)	Five log reductions of pathogenic microorganisms, PEF with preheating significantly reduce the enzyme activity	Horlacher et al. (2024)
High‐intensity pulsed electric fields Low intensity pulse electric field	Almond milk Pulse width (40 μs), electric field strength (7–28 kVcm−1: 200 μs)	Significantly reduce particle size, stability and clarity were improved	Manzoor et al. (2019)
	Soy milk; 2–10 kV/cm, 10, 55, and 100 monopolar pulses (4 μs width)	Increase β‐glucosidase enzyme, aglycones and isoflavone content	de la Morales‐ Peña et al. (2022)
High‐pressure processing	Oat milk; Pressure range (300 to 600 MPa)	Improved viscosity, color, protein content and effectively inactivate microorganisms at 600 MPa	Ahmad et al. (2025)
Cold plasma	Sesame milk; Power (25, 60 and 120 W), current (24.5, 40.3, and 59.8 mA)	Lipoxygenase activity and anti‐nutritional factor were reduce	Dharini et al. (2023)
Ultra high‐pressure homogenization	Almond milk; Heat at 72°C, 85°C, and 99°C pressure (10 min) at 150, 300, 450, and 600 MPa at 30°C	Amandin immunological reactivity and reduced solubility of proteins up to 70%	Dhakal et al. (2014)
	Tiger nut milk; Pressure 200 and 300 MPa, Temperature 40°C	Microbial inactivation rate higher than homogenized‐pasteurized process, shelf life increases up to 1 month	Codina‐Torrella et al. (2018)
High pressure homogenization with pH shift	Hemp milk; After a thorough washing, the hemp seeds were mashed in five volumes of deionized water using a blender set to 12,000 rpm for 3 min. After filtering the mixture, it was homogenized twice at 30 or 60 MPa. Before or after homogenization, the milk was adjusted to pH 12 using 2 N NaOH for the pH shift process. The raw hemp milks were made by mixing without changing the pH or HPH	The pH shift + HPH showed slow down the hydroperoxides and malondialdehyde production, but also reduce microbial population in hemp milk	Wang et al. (2018)
UV technology	Soy milk subjected to UV doses: 0–10 J/cm^2^ and different temperatures (4°C, 8°C, 12°C, 18°C, 25°C and 30°C)	The result demonstrated that UV inactivation was temperature dependent and that increasing the temperature from 4°C to 18°C increased inactivation efficiency of microbes	Possas et al. (2018)

High‐pressure processing, sometimes called HHP, makes use of pressures (100–800 MPa), sometimes along with heat (Balasubramaniam et al. 2015). When applied to food, the combined effect of heat and pressure may change its physicochemical properties in several ways. The sort of food determines whether a batch or semi‐continuous procedure is best suited for HHP. Using HHP changes milk's texture, sensory qualities, and nutritional value while modifying its features, particularly its proteins. (Bernat et al. 2015) investigated the influence of heat treatment and high‐pressure homogenization (HPH) on the size of the particles of hazelnut and almond milk, both separately and together. Thermal treated liquids had a larger volume mean diameter than untreated beverages. The use of HPH and thermal treatment helped to control the size of the particles that were suspended between the two liquids. It was found that a higher volume was linked to an increase in temperature in the combined treatment, lending credence to the idea that almond and hazelnut beverages can be stabilized by the application of HPH, followed by low heat treatment. A combination of effective times and temperatures acts as a way to lessen influence of thermal processing on the standards of PBBs. Pasteurization within 100°C, sterilization to 20 min at 121°C, and UHT ranging from 135°C to 150°C have all been tried thus far (Sethi et al. 2016). Another study focused on the physical and oxidative stability of hemp milk made without additives using the pH shift and HPH procedure. In an effort to preserve the final product's original flavor and palatability, emulsifiers and heat treatments were not employed. Protein interaction was encouraged by the pH shift treatment before HPH, leading to the creation of significant masses and aggregates that stabilized the emulsion. Moderate aggregation of protein generated a sterile stabilization of oil droplets from oxidative agents, as evidenced by the fact that hemp milk with such interaction structures was more resistant to oxidation and durable against coalescence. Additionally, the pH change and HPH together were highly detrimental to microorganisms and guaranteed a small number of microbes in hemp milk while it was refrigerated (Wang et al. 2018). When soymilk enriched with calcium was processed with HHP, a comparable result was noted (Manassero et al. 2016). Numerous bacteria can benefit from the abundance of nutrients included in PBBs, which may affect the safety and quality of the product while it is being stored. The application of UHPH as a promising technique to extend the shelf life of PBBs is supported by numerous studies. The primary benefit is that the microbial load can be partially or completely inactivated, increasing the shelf life of PBBs. For example, the microbial load in tiger nut milk was decreased by UHPH (200 and 300 MPa, 40°C), especially Enterobacteriaceae, Lactobacillus, molds, and yeasts (Codina‐Torrella et al. 2018).

Innovative thermal treatments distinguish themselves from traditional pasteurization techniques, particularly in terms of their capacity to disperse heat throughout the systems. By applying energy in a volumetric fashion, innovative thermal treatments lessen the impacts of thermal deterioration on the thermolabile chemicals in products. Otherwise, the challenges of designing these kinds of equipment and the initial investment expenses must be considered when assessing the use of emerging technologies in large‐scale industrial processes. Despite these challenges, developing technologies are linked to great energy efficiency because they enable quick, low‐energy operations. Every new technology has its own cost–benefit analysis for various tools and pasteurization processes (Chiozzi et al. 2022). In PEF, Long‐term operational difficulties include corrosion of electrodes and maintenance problems, which call for regular replacements and cleaning to preserve processing effectiveness (Pataro and Ferrari 2020). Due to scaling issues, ultrasonication's industrial application is still restricted, despite its promising results at the laboratory and pilot stages. For large‐scale application, high‐power ultrasound equipment that can effectively process huge amounts of food must be developed (Akhi et al. 2025; Tufail et al. 2025a, 2025b). Obtaining regulatory approval and implementing cold plasma in the plant‐based food industry are difficult tasks. A substantial amount of testing with large apparatus is necessary for the approval procedure, which comes with high installation expenses (Yepez et al. 2022). In industrial processes, maintaining uniformity and reproducibility continues to be crucial. In order to improve these techniques and encourage their broad application, interdisciplinary cooperation between food scientists, researchers, and business executives will be essential going forward. The advancement of economical processing methods, the incorporation of real‐time monitoring systems, and the combination of several non‐thermal procedures for increased efficiency should be the top priorities for research. Additionally, commercial acceptability will be facilitated by resolving regulatory issues and educating customers about the advantages and safety of these technologies.

## 7 Health Concerns of PBBs

The raw ingredients used in the production of PBBs pose a number of safety risks. Antinutrients, microbes, mycotoxins, and the possible allergic effects of specific ingredients are some of these risks.

### 7.1 Antinutritional Profile

Antinutrients found in plant raw materials, such as phytates and oxalates in seeds and nuts, are able to enter plant‐based beverages and cause several adverse impacts, such as decreased absorption of certain nutrients. Furthermore, certain production processes, such as soaking and thermal processing, can reduce or minimize the quantity of antinutrients in beverages and lessen their antinutritional qualities (Hidalgo‐Fuentes et al. 2024). Significant levels of oxalates are found in almonds, cashew nuts, and other nuts; these compounds absorb into drinks and prevent the absorption of calcium. Additionally, they have the ability to combine with calcium to produce a complex that can lead to kidney stones (Chalupa‐Krebzdak et al. 2018). Saponins, which are glucosides found in legumes and pseudo‐cereals, might be regarded as antinutrients, as they obstruct the absorption of proteins, particularly soy proteins, by forming insoluble saponin–protein complexes that are difficult to break down. They may also show signs of hemolysis (Manzoor et al. 2021). Lectins, which are frequently present in soy and other grains, are another type of antinutrient present in plant‐based beverages. They have an impact on how food is used for energy and significantly slow down the intestinal absorption of glucose (Santiago et al. 1993). Proteins or glycoproteins called lectins and hemagglutinins include at least one noncatalytic domain, which allows them to bind reversibly to monosaccharides or oligosaccharides in particular. Additionally, they can cause erythrocyte agglutination by binding carbohydrate residues on the surface of erythrocytes (Manzoor et al. 2021). Plants frequently include protease inhibitors, which are becoming more and more significant in study because they significantly lower enzyme activity through protein–protein interactions. Protease inhibitors (trypsin and chymotrypsin) are usually present in legumes. Protein digestion is inhibited as a result of their interference with the intestinal trypsin and chymotrypsin enzymes. Plant serpins, a family of important protease inhibitors, are mostly found in cereal seeds. Serpins are strong trypsin and chymotrypsin inhibitors (Rachtan‐Janicka et al. 2025).

### 7.2 Microbiological and Mycotoxin Contamination

PBBs that are sold commercially undergo heat treatment until they are considered microbe‐free. Still, there is a chance that the final product will be contaminated (Gützkow et al. 2024). It was demonstrated that pathogens including *Listeria monocytogenes* and *Salmonella* spp. may thrive and proliferate in PBBs under experimental conditions at temperatures (4°C, 8°C, and 20°C) despite the application of heat procedures (Bartula et al. 2023). PBB may encourage the growth of bacteria from a microbiological perspective. They comprise free sugars like fructose and glucose, which are digested by various kinds of bacteria and can cause a significant rise in the microbial population linked to sensory loss or, in some cases, foodborne illness (Gützkow et al. 2024). It is very challenging to eradicate spore‐forming bacteria from food materials or in food production plants, such as *Bacillus* and *Clostridium*. A botulism case linked to almond beverage intake demonstrates the need for a deeper understanding of potential risks *C. botulinum* poses to food safety (Rachtan‐Janicka et al. 2025).

Filamentous fungi can infect raw agricultural items like grains, legumes, and nuts, increasing the possibility of mycotoxins in processed meals like PBBs. When ingested in large quantities or continuously, they are detrimental to health (Pavlenko et al. 2024). Beverages made from nuts and oats, especially those manufactured from almonds, had the greatest mycotoxin contents. Tentoxin, ENNB, and ENNB1 concentrations in almond‐based beverages varied from 15 to 98 μg/L, 10 to 109 μg/L, and 6 to 60 μg/L, respectively. On the other hand, tentoxin, ENNs, ZEA, and HT‐2 toxin showed the highest quantities in oat beverages (Juan et al. 2022; Miró‐Abella et al. 2017). There is currently inadequate data on this kind of exposure from consumption of PBB. (Pavlenko et al. 2024) computed the probable daily intake (PDI) values based on the supposition that PBMS are most frequently added to tea and coffee. The PDI values ranged from 0.01 to 7.62 ng/kg and 0.01 to 4.29 ng/kg body weight per day (bw/day) across all age groups of males (19 and 39) and females (19–34) who had been exposed to nivalenol had the highest levels of mycotoxin exposure. Based on typical levels of contamination in nut‐based beverages, aflatoxin B1 was shown to have the lowest value. Based on simulations of PDI values for individuals in Northern Europe, the average PDI concentrations for zearalenone (0.00149 μg/kg), deoxynivalenol (0.48282 μg/kg), and ochratoxin A (0.6764 μg/kg) bw/day were calculated. Therefore, in comparison with these simulated values, it may be stated that the presence of mycotoxin contamination in PBBs is extremely small. Overall, Pavlenko et al. (2024) reported that all of the PBBs evaluated were safe for consumption by consumers based on the maximum contamination levels.

### 7.3 Allergens

Serving PBBs to people who may be allergic to the proteins in nuts, cereals, and legumes should be handled with precaution. In a vegan diet, legumes are a vital source of protein. They have also been known to trigger allergic responses. The frequency of legume protein allergies is influenced by a number of variables, such as dietary practices and geographic location. For instance, allergies to chickpea proteins are more prevalent in India and to lentil proteins in Mediterranean nations (Sharma et al. 2022). Soy allergies are mostly caused by storage proteins (11S glycine and ß‐conglycinin), which are associated with serious allergic responses (Kern et al. 2019). A protein called PR‐10 for those who are allergic to birch pollen, Gly m 4 can result in oral allergy condition, which can include severe anaphylactic reactions when cofactors are present or after ingesting soy beverages. This is responsible for both the high prevalence of PR‐10 hypersensitivity and increased consumption of soy beverage in place of cow's milk (Präger et al. 2023).

The WHO/IUIS Subcommittee on Allergen Nomenclature's allergen database lists 18 peanut allergens (WHO/IUIS Allergen Nomenclature Subcommittee n.d.). The storage proteins in peanuts (Ara h 1, Ara h 2, and Ara h 3) appear to be responsible for the most severe allergies.

People who are allergic to peanuts, other legumes, and tree nuts frequently experience cross‐reactions since allergens from these three groups have been found to be important allergens in other legumes and tree nuts.

The primary protein that causes almond allergies is amandine. (Devnani et al. 2020; Dhakal et al. 2014) reported reduced quantities of protein, including amandine, in almond beverages processed under high pressure and heat. On the other hand, studies have revealed the existence of heat‐stable allergens that might cause allergy symptoms (Mandalari and Mackie 2018); as a result, people who are allergic to almonds should avoid eating anything manufactured from or containing almonds. Furthermore, cooking has no effect on allergenicity since the primary allergenic components in pea proteins are heat tolerant. There are seven known allergies in peas: Agglutinin, albumin, Bet v1 homolog (Pis s 6), storage protein (Pis s 1 and Pis s 2), lipid transfer protein (Pis s 3), and profilin (Pis s 5). There have been reports of cross‐reactivity with chickpeas, lentils, and peanuts (Präger et al. 2023).

## 8 Labeling of PBBs

PBBs are classified as nonalcoholic beverages under Part E of Annex II to Regulation (EC) No 1333/2008 (European Parliament and Council of the European Union 2008). Today, consumers are informed about the features of a food product through food labeling and advertising in compliance with EU Regulation 1169/2011 (European Parliament, and Council of the European Union 2011), particularly through the nutrition and health claims found in the product (European Parliament and Council of the European Union 2006). These details are not required to appear on food labels; if they are included, then any claims should be precise, understandable, and supported by scientific data. Customers often assume that products featuring claims on their labels are healthier than those without such claims, and they frequently ascribe greater functional or nutritional advantages to these products than the claims themselves (Tønnesen et al. 2022). The term “milks” was formerly used to describe PBBs that were consumed in place of milk. According to the (European Parliament and Council of the European Union 2013), this word has been deemed inaccurate and deceptive to consumers in Europe. Nonetheless, an order issued by the European Union Commission (Court of Justice of the European Union 2017) permits certain PBBs that are customarily referred to as “almond milk” (Spain) or “coconut milk” (Portugal) to be exempt from the term “milk.” Due to market demand from the dairy sector to differentiate products, the name “mylk,” which is derived from “milk,” is used for PBBs in other nations (Silva and Smetana 2022). The Court of Justice of the EU ruled on June 14, 2017, confirming the definition notwithstanding the protests against this exclusive classification of milk (Council of the European Union 2007).

It is recommended to include allergen information on food labels, including those for PBBs should be considered (European Parliament, and Council of the European Union 2011). Annex II to Reg. 1169/2011 (European Parliament, and Council of the European Union 2011) contains a list of substances, products, and their derivatives that can trigger food allergy reactions. Allergens found in PBBs include soy‐based products, tree nuts and peanuts, and gluten‐containing cereals like wheat, barley, and oats. Information regarding the ingredient that causes allergies or intolerance reactions must be listed on the label by the manufacturer. The name of the ingredient must be emphasized in a typeface that distinguishes it from the list of other substances in the finished product, such as by design, size, or background color.

The consumer must have easy access to, clear visibility of, and readable information about allergies (Rachtan‐Janicka et al. 2025). The fact that the labeling complied with EU Regulation 1169/2011 regarding nutritional information was one of the inclusion criteria. This means that all of the required information (energy value, fat, saturated fatty acids, carbohydrates, sugars, proteins, and salt) should be included in the same visual chart and expressed per 100 g or 100 mL. Mono and polyunsaturated fatty acids, polyols, starch, fiber, vitamins, and/or minerals are optional additions to this required information (but only if the products provide more than a significant amount, such as 7.5% of Nutrient Reference Values, as defined in EU Regulations) (Pérez‐Rodríguez et al. 2023).

## 9 Sensory Studied of PBBs

Human sensory perception is crucial to the manufacturing of PBBs since sensory qualities influence consumer acceptance of the product (Zandona 2021). In general, the sensory analysis of PBBs has shown that this type of product lacks taste and odor/flavor. In this context, (Cardello et al. 2022) divided into four groups of 345 New Zealand consumers according to whether they preferred samples of plant‐based or cow's milk. The findings demonstrated that among various customer segments, sensory characteristics, particularly flavor, were more powerful determinants of liking (Jaeger et al. 2023; Moss et al. 2023; Pramudya et al. 2019).

Numerous researches outlined the sensory qualities of legume‐based milk and emphasized how their grassy and beany aromas and scents are influenced by n‐hexanal and n‐hexanol from lipid oxidation in plants (Tangyu et al. 2019). Lawrence et al. (2016) investigated the sensory aspects that influence 235 US customers' preference for 12 commercial unflavored soymilks. The findings indicated that the taste and flavor of the soymilk samples had the biggest impact on the overall preference. Acquiring a similar approach, Vaikma et al. (2021) assessed 90 PBBs and created a sensory map of them. Cereal and pseudo cereal beverages featured cereal odor and flavor, with oats exhibiting a stronger aftertaste, whereas legume‐based beverages (soy) demonstrated a stronger legume odor and flavor. Seed‐based milk (hemp beverage) smelled like hay, whereas nut‐based milk substitutes had a nutty flavor. Nut and seed‐based beverages have unpleasant off tastes (Vaikma et al. 2021). Pramudya et al. (2019) assessed the rice‐based milk substitutes, discovering 23 sensory characteristics and figuring out which have an impact on the products' customer acceptability. Particularly, higher scorings for sweetness, astringency, nutty, and grainy flavors are linked to better overall liking, while higher ratings for dark color, yeasty/fermented, starchy scents, and woody odors, bitter and sour sensations were connected with lower overall liking. Most intriguing were the vanillin characteristics: samples' overall liking appeared positively impacted by the flavor (retro‐nasal smells), while the aroma (orthonasal odors) was negatively correlated. The authors noted that retronasal odors may enhance sweetness, while orthonasal odors may be an unfamiliar and unexpected feature (Proserpio et al. 2021).

PBBs have been described as having bitter off‐tastes, sour tastes, and metallic or astringent sensations when it comes to taste and tactile sensations. These sensations are associated with the presence of chemicals like phenols, terpenes, etc. (Tangyu et al. 2019). When two studies (Gorman et al. 2021; Chung et al. 2022) sought to determine the sensory factors that influence consumers' preferences for plant‐based milk coffees. The findings indicated that the more comparable a plant‐based milk coffee was to dairy milk coffee; the more favorable sensory experiences were thought to be. The coffee samples' acceptability was undermined by strong, grassy, rancid oil, earthy, and beany aromas and sensations like sour, oily, and astringent. To solve the sensory problems, thermal processing on the PBBs could be utilized to get rid of the bad flavors and tastes (Reyes‐Jurado et al. 2023). Additionally, PBBs may be flavored (Moss et al. 2022) or sweetened with sugars to promote consumer acceptance (Aydar et al. 2020; Reyes‐Jurado et al. 2023). In the research suggested by (Moss et al. 2022), the general acceptance of PBBs that were flavored (vanilla and chocolate) and unflavored (almond and oat) was assessed. The addition of the chocolate and vanilla taste considerably raised the customer's like scores.

PBBs can also have problems with texture or mouthfeel; the presence of large particle aggregates may cause a chalky, grainy, or gritty sensation (Tangyu et al. 2019). Waterier textures are typically found in cereal and pseudocereal beverages (Pramudya et al. 2019), whereas beverages made with nuts could have a thicker, lumpier texture (Vaikma et al. 2021). Blending various PBBs is another method for overcoming off‐flavors. In a research done in Ghana by Oduro et al. (2021), using a mapping method, advancements in three‐blend plant‐based milk formulations manufactured from raw plant materials (melon seeds, peanuts, coconuts, and tiger nuts) were discovered. Furthermore, sweetened UHT milk and commercial soymilk were also added. As anticipated, consumers preferred the sweetened UHT milk and soymilk over the modified PBBs. However, it was shown that the products made using melon seed milk were the least appealing. However, the products containing melon seed milk were found to have the lowest acceptability, while two blended samples: Sample 1, containing Tiger nuts, Coconuts, and Peanuts in proportions of 37.5%, 25%, and 37.5%; and Sample 2, with Tiger nuts, Coconuts, and Peanuts at 25%, 50%, and 25% respectively have been demonstrated to have a good possibility of being accepted by customers. More research is required because there is a dearth of latest scientific literature on blending of different PBBs or because it raises serious problems with the experimental approach.

## 10 Sustainability of PBBs

There is increasing evidence that present world systems of agriculture and patterns of consumption are not appropriate for human health (Willett et al. 2019). If consumers do not implement significant changes in eating habits, it seems doubtful that agricultural industries will be able to fulfill the world's environmental goals. Nevertheless, numerous opportunities throughout the food system might bring about significant change. There is a global trend to switch to sustainable diets that are high in plant‐derived and whole foods and low in animal‐derived food in order to promote simultaneously individual well‐being and environmental sustainability (Alae‐Carew et al. 2022). PBBs may have an advantage over traditional dairy products because of their marketing, which usually highlights sustainability, a commitment to environmental safety, eliminating unnatural foods, or the humane treatment of animals (Schiano et al. 2020). McCarthy et al. (2017) conducted a means‐end‐chain interview with consumers who purchase dairy and nondairy goods as part of a conjoint study. They found that among nondairy customers, the plant based characteristic elicited ethical judgments according to environmental consequences and animal care, creating a value ladder. However, Pua et al. (2022) draw attention to examining a wider range of PBBs to provide better‐tasting and more nutritious products. As reported by Pelletier et al. (2013), younger people who emphasis the safe production of food often ate better. This implies a possible connection between sustainability concepts and a balanced diet. This research suggests that there may be a positive correlation between perceptions of good health and sustainability, a phenomenon known as the halo effect. Consumer values of conservation of resources, well‐being, and transparency meet when it comes to natural goods (Aschemann‐Witzel 2015). Studied has demonstrated that substituting animal products with plant‐based foods will greatly reduce the negative effects on the environment as well as the prevalence and mortality rate of non‐communicable diseases (Alae‐Carew et al. 2022). PBBs are frequently promoted as a sustainable substitute for dairy milk that (Präger et al. 2023) has lessen the environmental effect of consumers' purchases. PBBs under the Silk brand, for instance, claim that their beverages are less carbon footprint than traditional dairy milk in the US (Berardy et al. 2022). The search for more sustainable food production chains is sparked by severe concerns about climate change and the world's expanding population (Parmesan et al. 2022). The so‐called “protein transition,” in which plant based proteins take the place of animal‐based proteins, is one example. One measure of sustainability improvement is greenhouse gas emissions (GHGs), or CO2‐eq per kilogram of product. Alternatives derived from plants typically have less environmental impact. Dairy substitutes including soy, oat, and almond beverages, for instance, are said to have a substantially smaller environmental impact than milk (Clune et al. 2017). It should be mentioned that this study compares (nonfortified) milk, fortified PBBs, and PBBs solely on the basis of their primary plant‐based ingredients, such as soy and oats, to assess the carbon footprint of these products (de Jong et al. 2024).

Researchers are using life cycle assessment (LCA) to evaluate the environmental impact of food systems, especially milk, because of the need for environmentally sustainable food production. LCA is a highly versatile method to evaluate the environmental effect of any product or service and suggest areas for improvement (Goucher et al. 2017; Yadav et al. 2023). The significant benefits of LCA are its extensive coverage of environmental consequences, and it helps to grasp the full issue to find a set of optimal approaches (Baldini et al. 2017). Previous studied reported that PBBs generates less Global warming potential (GWP) than animal milk. Among animal milks, cow milk has the lowest mean GWP (1.29 kg CO2 eq. per kg) than other mammalians milk. Among all milk varieties, coconut milk has the lowest mean GWP (0.257 kg CO2 eq), followed by oat, soy, pea, and almond. The process of producing PBBs is comparatively greener. According to the analysis, all PBBs have GWP values < one, while animal milk has GWP greater than 1 kg CO2 eq (Khanpit et al. 2024). Another key impact measure is the water footprint, showing the quantity of water consumed during cultivation for animal feed and PBBs raw material. Animal milk, on the other hand, uses relatively less water. It was found that in the first 2 years, almond trees flourishes without any crop output, and the production increases progressively until Year 7, when it attains a steady yield (Winans et al. 2020). The water footprint of California almonds is 10,240 L/kg of kernel (Fulton et al. 2019). Khanpit et al. (2024) demonstrate that the spectrum of water footprint is varied. One potential reason is the choosing of water footprint effect indicator. Henderson and Unnasch (2017) used blue, green, and gray water, and the water scarcity factor (WSF) produced a water footprint of roughly 33,153 L per FU, whereas Winans et al. (2020) used fresh water usage, or 175–183 L of water per FU. If WSF is taken into account, the water footprint of almond, pea, and oats are much higher than animal milk. Incorporating WSF overstates actual water use; consequently, it is appropriate to remove WSF in computing water footprint, which is what we did. The average water footprint (PBBs and milk) is as follows, in ascending order, based on a comparison of buffalo > oat > pea > almond > cow> coconut (Khanpit et al. 2024). Another important but largely unstudied midway indicator in milk systems is land use. The agricultural area needed to grow crops for animal feed is the primary source of land use impact for the animal milk system. The land usage (m^2^/kg milk) for sheep, goat, camel, almond, and coconut milk was not examined in any of the analyzed studies; this is a possible subject for future research (Khanpit et al. 2024). PBBs are more sustainable than mammalian milk in terms of GWP and land use, while mammalian milk is more energy efficient and has a lower water footprint than PBBs. When water shortage is taken into account, PBBs have a significantly higher water footprint than mammalian milk.

## 11 Conclusion and Future Research

Society's problems with climate change and its effects on food security are mostly related to the food system. These are clear indications that the food sector needs to change quickly to adopt sustainable methods of producing food. PBBs growing market demand catalyzes more product development. PBBs play an important part in human health for consumers who are influenced by factors like allergies, and consumers who select this particular kind of product out of conviction. Research is focused on retaining the bioavailability of nutrients during storage and fortifying PBBs with a suitable kind of fortifier using adequate technology to make it possible to use it as a nutritionally equivalent alternative for bovine milk. Evaluating the safety and shelf life of PBBs substitutes and testing for allergies to guarantee the safety of novel and new ingredients. Future research would be conducted on the possible long‐term health effects of switching from dairy to PBBs and nanotechnology fortification, flavor‐masking, clean‐label trends, circular economy, and digital consumer engagement. The assessment of the sustainability of milk and PBBs that incorporate nutrients, as well as their bioactivity, has demonstrated the need for an in‐depth investigation of this matter.

## Author Contributions

**Nabeel Ashraf:** writing original draft and data curation. **Zunaira Arshad:** writing original draft and data curation.**Ahmad Din:** supervision. **Huma Bader Ul Ain:** methodology. **Esther Ugo Alum:** conceptualization. **Tabussam Tufail:** writing, reviewing, and editing and supervision.

## Funding

This study was supported by the “Pioneer” and “Leading Goose” R&D Program of Zhejiang (No. 2023C03038) and the National Natural Science Foundation of China (No. 82374017).

## Ethics Statement

The authors have nothing to report.

## Conflicts of Interest

The authors declare no conflicts of interest.

## Data Availability

The authors have nothing to report.
